# Characterization of DNA Binding Property of the HIV-1 Host Factor and Tumor Suppressor Protein Integrase Interactor 1 (INI1/hSNF5)

**DOI:** 10.1371/journal.pone.0066581

**Published:** 2013-07-04

**Authors:** Supratik Das, Baisakhi Banerjee, Maidul Hossain, Muruganandan Thangamuniyandi, Saumya Dasgupta, Nipa Chongdar, Gopinatha Suresh Kumar, Gautam Basu

**Affiliations:** 1 Department of Biochemistry, University of Calcutta, Kolkata, India; 2 Dr. B.C. Guha Center for Genetic Engineering and Biotechnology, University of Calcutta, Kolkata, India; 3 Biophysical Chemistry Laboratory, CSIR-Indian Institute of Chemical Biology, Kolkata, India; 4 Central Instrumentation, CSIR-Indian Institute of Chemical Biology, Kolkata, India; 5 Department of Biophysics, Bose Institute, Kolkata, India; Ohio State University, United States of America

## Abstract

Integrase Interactor 1 (INI1/hSNF5) is a component of the hSWI/SNF chromatin remodeling complex. The *INI1* gene is either deleted or mutated in rhabdoid cancers like ATRT (Atypical terratoid and rhabdoid tumor). INI1 is also a host factor for HIV-1 replication. INI1 binds DNA non-specifically. However, the mechanism of DNA binding and its biological role are unknown. From agarose gel retardation assay (AGRA), Ni-NTA pull-down and atomic force microscopy (AFM) studies we show that amino acids 105–183 of INI1 comprise the minimal DNA binding domain (DBD). The INI1 DBD is absent in plants and in yeast SNF5. It is present in *Caenorhabditis elegans* SNF5, *Drosophila melanogaster* homologue SNR1 and is a highly conserved domain in vertebrates. The DNA binding property of this domain in SNR1, that is only 58% identical to INI1/hSNF5, is conserved. Analytical ultracentrifugation studies of INI1 DBD and INI1 DBD:DNA complexes at different concentrations show that the DBD exists as a monomer at low protein concentration and two molecules of monomer binds one molecule of DNA. At high protein concentration, it exists as a dimer and binds two DNA molecules. Furthermore, isothermal calorimetry (ITC) experiments demonstrate that the DBD monomer binds DNA with a stoichiometry (N) of ∼0.5 and K_d_  = 0.94 µM whereas the DBD dimer binds two DNA molecules sequentially with K’_d1_ = 222 µM and K’_d2_ = 1.16 µM. Monomeric DBD binding to DNA is enthalpy driven (ΔH = –29.9 KJ/mole). Dimeric DBD binding to DNA is sequential with the first binding event driven by positive entropy (ΔH’_1_ = 115.7 KJ/mole, TΔS’_1_ = 136.8 KJ/mole) and the second binding event driven by negative enthalpy (ΔH’_2_ = –106.3 KJ/mole, TΔS’_2_ = –75.7 KJ/mole). Our model for INI1 DBD binding to DNA provides new insights into the mechanism of DNA binding by INI1.

## Introduction

The SWI/SNF chromatin remodeling complex in yeast is a large multisubunit complex, required for regulation of mating type switching, sucrose-dependent growth and transcription [1]. Counterparts of the SWI/SNF complex have been found in Drosophila and humans suggesting that this type of complex is conserved from yeast to mammals [2], [3], [4]. SWI1, the DNA-dependent ATPase SWI2/SNF2, SWI3, SNF5 and SNF6 are the core components of the yeast SWI/SNF complex [2]. The Drosophila homologue of SNF5 is SNR1 and the human homologue is INI1/hSNF5. INI1, SNR1 and SNF5 proteins show strong sequence similarity over a region comprising Repeat 1 (Rpt1), Repeat 2 (Rpt2) and Coiled-coil (CC) motifs [2], [3], [4], [5]. Biallelic deletion of the *INI1* gene and mutations - missense, nonsense, splicing and frameshift in INI1 have been implicated in pediatric cancers with poor prognosis like rhabdoid tumors and other cancers of the soft tissue [6]. The mechanism by which the loss or mutation of INI1 leads to cancer is not fully understood although cell culture experiments and studies in mice suggest that INI1 mediated transcription regulation of the cell cycle regulatory protein cyclin D1 maybe important [7], [8]. However, deletion or mutation of *INI1* can also potentially affect other functions of INI1, including its role through the SWI/SNF complex, as mutations throughout the gene have been found in patients with rhabdoid cancers. INI1 is also one of the host factors that regulate propagation of HIV-1 virus in infected cells. INI1 is involved in HIV-1 integration, one of the critical steps in HIV-1 replication. INI1 not only binds integrase (IN), a viral enzyme that is absolutely required for the integration of the reverse transcribed viral double stranded DNA into the host chromatin, but also modulates the activity of IN [9]. Earlier studies have shown that INI1 stimulates as well as inhibits IN activity depending on the concentration of IN and the ratio of IN:INI1 [10]. Furthermore, these studies demonstrate that INI1 is a multimer and that multimerization of INI1 is essential for its ability to bind IN to form a high MW complex that is required for inhibition of IN activity [10]. On the other hand, the ability of INI1 to stimulate IN activity appears to require a different mechanism that involves the DNA binding activity of INI1 [10]. Whether other components of the SWI/SNF complex play a role in INI1 function in integration is unknown. However, chromatin remodeling by INI1 containing SWI/SNF complex overcomes the barrier to full-site integration imposed by stable and regularly positioned nucleosomes [11]. INI1 exhibits non-specific DNA binding activity at the minor groove [10], a characteristic of transcription factors that are architectural proteins.

It is generally thought that promoters recruit the SWI/SNF complex via its interaction with DNA-binding factors. However, studies have also shown that the SWI/SNF complex binds DNA in a manner reminiscent of the HMG family of architectural proteins [12]. The complex was shown to interact with the minor groove of DNA, bind 4-way junction DNA and introduce positive supercoils in relaxed plasmid DNA [12]. It has been suggested that these properties of the SWI/SNF complex may play an important role in chromatin remodeling by this complex [12]. The components of the SWI/SNF complex contain different DNA binding motifs [13]. Some of the common DNA binding motifs present in components of the SWI/SNF complex from different species are HMG (High Mobility Group), ARID (AT-rich interaction domain) and SWIRM DNA binding motifs [13], [14], [15]. Among human SWI/SNF subunits, INI1/hSNF5 is known to bind DNA non-specifically at the minor groove [10] while hBRG1 has been reported to possess an AT-hook motif which appears to play a role in DNA binding [16].

The functional domains of INI1 are not well characterized except that Rpt1 and Rpt2 motifs are involved in protein-protein interaction, with both viral and cellular proteins. Although INI1 binds to DNA non-specifically and previous evidence suggests that DNA binding by INI1 may play an important role in stimulating HIV-1 integrase activity [10], the mechanism of DNA binding and its biological functions are unknown. Previously, using truncation mutants of INI1, Morozov et al [5] had shown that the region overlapping mutants D2 (amino acids 106–385) and 27B (amino acids 1–243) binds DNA. Since mutants 1.2 (amino acids 181–385) and 9.2 (amino acids 183–385) showed reduced DNA binding activity, it was suggested that the region, amino acids 106–183, in INI1, may be important for DNA binding activity [5]. However, there was no direct demonstration that this region in INI1 indeed has DNA binding activity. In this study, we have purified recombinant INI1 (105–183) and show directly that this region of INI1 indeed binds DNA. This domain is not present in plant SNF5 homologues and in yeast SNF5. It is present in the *Caenorhabditis elegans* SNF5 homologue, *Drosophila melanogaster* SNR1 and has evolved into a highly conserved domain in vertebrates. We further show that the homologous region in Drosophila SNR1 also has DNA binding property suggesting that the DNA binding property of this region in this family of proteins is phylogenetically conserved. The yield of purified recombinant full-length INI1 protein is low [10] making it hard to study the mechanism of DNA binding using biochemical and biophysical assays that require micromolar amounts of protein. This prompted us to use the purified recombinant INI1 DNA binding domain (DBD), which is obtained in high micromolar amounts, for mechanistic studies. Using analytical ultracentrifugation experiments, we show that the INI1 DBD undergoes concentration-dependent multimerization. At low concentration, the DBD is a monomer with two DBD monomer molecules binding one 22/23 nt U5 HIV-1 LTR DNA. At high concentration, the DBD is a dimer and it binds to two molecules of U5 HIV-1 LTR DNA, sequentially. Finally, using isothermal calorimetric experiments we have determined the stoichiometries, binding constants and thermodynamic parameters of DNA binding by the INI1 monomer and dimer. We demonstrate that the mode of DNA binding, and binding parameters for monomeric and dimeric INI1 DBD are different. The implications of these findings are discussed.

## Materials and Methods

### Plasmid DNA and Sequence Analysis

The region, amino acids 105–183 of INI1, was cloned into the *Nhe1*-*EcoR1* sites of pET28a to give pET28a-INI1DBD**.** The region, amino acids 90–167 of SNR1, was cloned into the *EcoR1*-*HindIII* site of pET28a to give pET28a-SNR1DBD**.** Presence of the putative INI1 DBD in INI1 homologues in different species was determined by non-redundant BLAST search and sequence conservation determined using the T-coffee server. Phylogenetic analysis of the DBD was carried out using MEGA 2.0 software.

### Purification of INI1 and SNR1 DBD

*E. coli* BL21DE3 competent cells were freshly transformed with either pET28a-INI1DBD or pET28a-SNR1DBD. Expressed protein was purified by Ni-NTA affinity chromatography and the fractions with the purest protein were pooled and dialyzed against buffer containing 20 mM HEPES (pH 7.2), 100 mM KCl, 10% glycerol, 0.1 mM EDTA, 1 mM DTT overnight at 4°C. Aliquots were made and stored at –80°C.

### HIV-1 LTR DNA

The double stranded (ds) HIV-1 LTR DNA was formed by annealing equimolar amounts of the TE buffer-dissolved oligos U5.4 (5′ ACTGCTAGAGATTTTCCGGATCC 3′) and U5.5 (5′ GGATCCGGAAAATCTCTAGCA 3′) in the presence of 100 mM NaCl by heating at 85–90°C for 5–10 min and then slow cooling at RT (room temperature). The amount of double stranded DNA obtained was measured in a Nanodrop 2000 spectrophotometer.

### AGRA

Agarose gel retardation assay was performed by incubating the desired amount of protein with about 100 ng of pET28a plasmid in buffer containing 20 mM HEPES (pH 7.2), 100 mM KCl, 5% glycerol, 0.1 mM EDTA, 1 mM DTT for 1 hour at 30°C. Complexes were resolved in a 1% agarose gel and DNA stained with ethidium bromide (EtBr).

### Ni-NTA Pull-down Assay

About 55 µM of either INI1 DBD (dimer) or SNR1 DBD (dimer) was incubated with 110 µM U5 HIV-1 LTR DNA in buffer containing 20 mM HEPES (pH 7.2), 100 mM KCl, 5% glycerol, 1 mM DTT for 30 min at RT and then put on ice. Ni-NTA beads, pre-washed with incubation buffer, were added to the protein-DNA reaction mixture and kept on ice for 1 hr with repeated mixing. Following incubation the beads were washed with incubation buffer containing 0.5% Triton-X three times for 5 min each. The protein-DNA complexes bound to the beads were divided into two parts and then analyzed by Western blot using α-His antibodies as probes to detect the His-tagged INI1 DBD and also by 10% urea-PAGE followed by ethidium bromide staining to detect bound DNA. For urea-PAGE analysis, the precipitated complex was heated in 90% formamide, 10% glycerol buffer for 10 min at 65°, quick chilled on ice and then separated in gels.

### Atomic Force Microscopy

Freshly cleaved muscovite Ruby mica sheet (ASTM V1 Grade Ruby Mica from MICAFAB, Chennai) was treated with APTES (3 Amino Propyl Tetra Ethylene silane) by vaporization method. Linearized vector DNA pNEB206A (NEB, Cat# N5502S) was used as a substrate. DNA alone (20 ng) or DNA:INI1 DBD complex (formed by incubating 50 ng of DNA with 75 nM INI1 DBD at RT for 30 min) were deposited on mica surface functionalized with APTES-mica in the presence of 25–250 nM MgCl_2_. Briefly, 10 µl of the DNA or DNA:protein solution were placed on APTES-mica surface for 5 min, rinsed with autoclaved, filtered, deionized water and vaccum dried under nitrogen gas. Atomic force microscopy (AFM) images were acquired in air using a Multi-Mode SPM Nanoscope IV system (Veeco, Santa Barbara, CA) operating in tapping mode. AAC mode AFM was performed using a Pico plus 5500 ILM AFM (Agilent Technologies USA) with a piezoscanner maximum range of 9 µm. Micro fabricated silicon cantilevers of 225 µm in length with a nominal spring force constant of 21–98 N/m were used from Nano sensors, USA. Cantilever oscillation frequency was tuned into resonance frequency. The cantilever resonance frequency was 150–300 kHz. The images (256 by 256 pixels) were captured with a scan size of between 0.5 and 5 µm at the scan speed rate of 0.5 lines/S. Images were processed by flatten using Pico view1.1 version software (Agilent Technologies, USA). Image manipulation has been done through Pico Image Advanced version software (Agilent Technologies, USA).

### Analytical Ultracentrifugation

Sedimentation equilibrium (SE) experiments were performed in a Beckman-Coulter XL-I analytical ultracentrifuge at 4°C using a 6-channel centerpiece placed in an AN-50 Ti rotor spun at speeds between 13,000 to 28,000 rpm. The INI1 DBD solution was run at two different concentrations of 10 µM and 60 µM (monomer). The U5 HIV-1 LTR DNA was run either alone or in complex with INI1 DBD at 1∶1 molar ratio of protein:DNA. The protein and protein:DNA complexes were formed in buffer containing 20 mM HEPES-KOH (pH 7.2), 100 mM KCl, 5–7% glycerol and 1 mM DTT by incubation at RT for 30 min. Absorbance scans were taken at 280 nm for protein and 260 nm for DNA or protein:DNA complexes at intervals of 5 hours till equilibrium was reached. The protein:DNA complexes were diluted such that A_260_ remained between 0.2–0.6. At these dilutions, the contribution of the protein to A_260_ was found to be not significant. The solvent density (1.014–1.026 g/ml) and partial specific volume (0.7154 ml/gm) of INI1 DBD were calculated using SEDNTERP. The partial specific volume of DNA with around 40% GC content is about 0.59 ml/gm [17]. The partial specific volume of U5 HIV-1 LTR DNA (47–48% GC content) was taken to be about 0.59 ml/gm. The partial specific volume of the protein:DNA complex was determined as described [17] and comes to 0.6478 ml/gm and 0.6691 ml/gm for monomer:DNA and dimer:DNA complexes. The extinction coefficients for protein and DNA were determined to be 8480 M^−1^ cm^−1^ and 568200 M^−1^ cm^−1^. The data was acquired in SEDFIT v14.1 and the molecular mass of the protein, DNA and protein:DNA complexes were determined in SEDPHAT v10.55b using species analysis model and global best fit.

### Isothermal Calorimetry

Isothermal titration calorimetry (ITC) experiments with 10 µM INI1 DBD (monomer) were performed on a Microcal VP-ITC microcalorimeter (MicroCal, Inc., Northampton, MA, USA) at 25°C. Aliquots of U5 HIV-1 LTR DNA (10 µL each from a stock of 55 µM) were injected from a 299 µL rotating syringe (611 rpm) into the isothermal sample chamber equilibrated at 25°C containing 1.4235 ml of INI1 DBD solution. Corresponding control experiments to determine the heat of dilution of the protein was also performed. The data were imported to Origin 7.0 and area under each peak was determined by integration as a function of time to give the measure of the heat associated with the injection. The heat of dilution was subtracted from the heat associated with mixing of protein and DNA to get the heat of binding for each injection. The resulting data were analyzed using Origin software to estimate the binding affinity (*K*), the enthalpy of binding (Δ*H*) and entropy of binding (Δ*S*). The free energies (Δ*G*) were calculated using the standard relationship, Δ*G* = Δ*H*–*T*Δ*S.* ITC experiments with 30 µM INI1 DBD (dimer) were performed on a Microcal ITC-200 microcalorimeter at 25°C using aliquots of U5 HIV-1 LTR DNA (1.4 µl each from a stock of 325 µM) and injected into 200 µl of INI1 DBD solution in the chamber. Control experiments and data analysis were done as described above.

## Results

### Conservation of the Region, Amino Acids 105–183, in INI1/hSNF5

**Figure 1:**
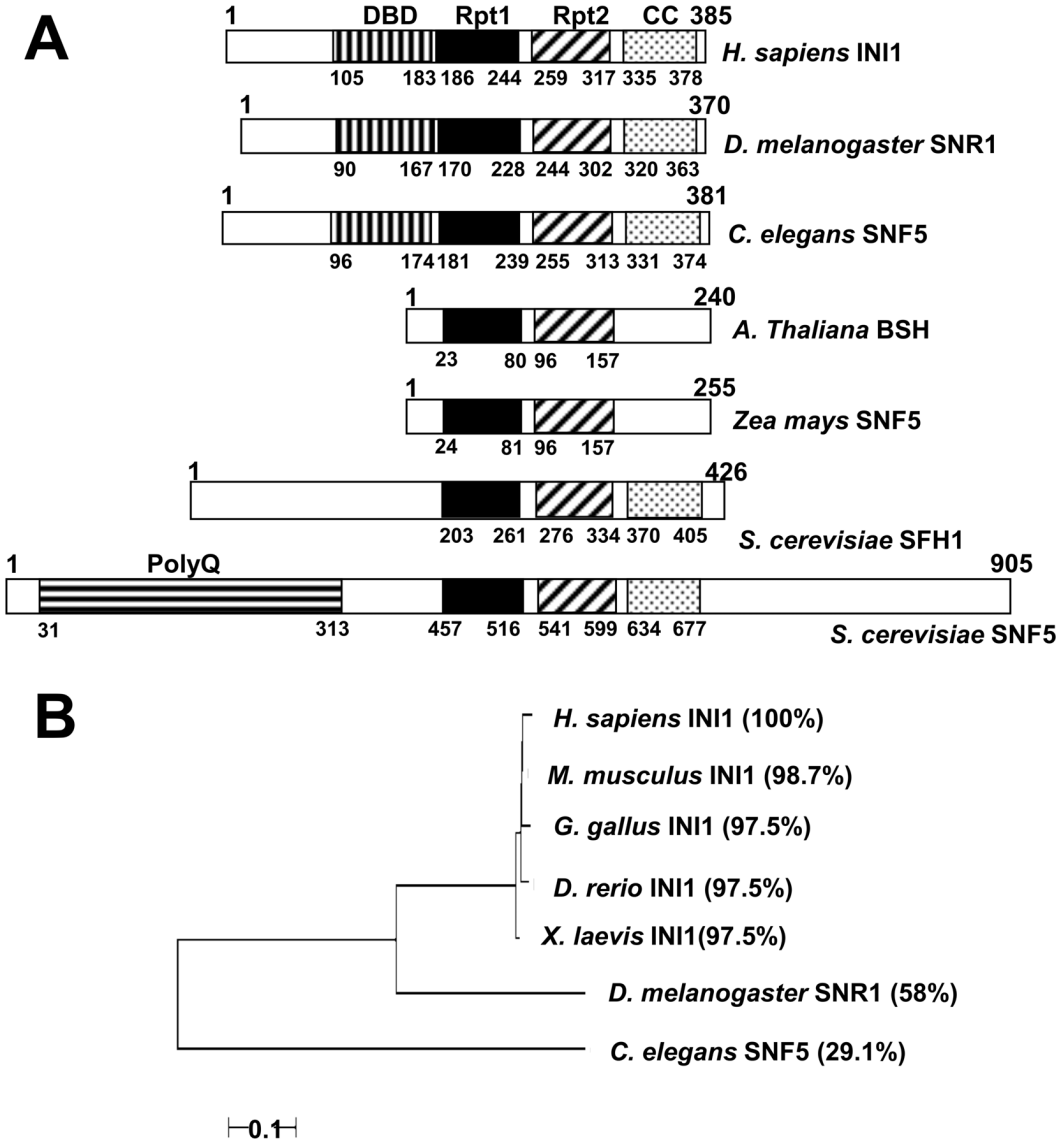
Phylogenetic conservation of the region corresponding to amino acids 105-183 of INI1. (A) Scheme showing presence and absence of different domains in INI1 from yeast to humans. (B) Neighbor-joining tree using MEGA 2.0 software showing conservation of the region corresponding to amino acids 105-183 of INI1. Percentage identity of the region in different species is shown in parenthesis.

Previous studies, using deletion mutants of GST-INI1 [5], have suggested that the region comprising amino acids 106–183 of human INI1 may be important for non-specific DNA binding activity. Studies carried out with the hydroxylapatite eluate of the INI1 multimerization-defective mutant I268T showed a partial defect (∼50% of wild type activity) in its ability to stimulate integrase activity [10] and also showed a partial defect in its ability to bind acceptor plasmid DNA [10] suggesting that DNA binding by INI1 may have biological functions. The I268T mutation lies in a region at the N-terminus of the Rpt2 motif of INI1 overlapping the nuclear export sequence (NES) [10]. This prompted us to look at the DNA binding property of INI1 in more detail. We found that the region comprising amino acids 105–183 of human INI1 is highly conserved in higher eukaryotes (Figure 1A). However, this region is absent in plant SNF5 like *Arabidopsis thaliana* (BSH), and *Zea mays* (SNF5) and the yeast *Saccharomyces cerevisiae* (paralogs SFH1 and SNF5). Phylogenetic analysis of this domain across different species of higher eukaryotes by neighbor-joining tree analysis and identity studies (Figure 1B) show that the region is highly conserved between human, mouse, chicken, frog and fish INI1 (Figure 1B). The conservation among these species lies between 97–99% identity (Figure 1B). However, as one moves down the phylogenetic order, sequence identity decreases to ∼58% in *D. melanogaster* SNR1 and ∼30% in *C. elegans* SNF5 (Figure 1B). When the other prominent domains in INI1 *viz* Rpt1 and Rpt2 are considered, it was found that these domains are present in both lower eukaryotes and higher eukaryotes while the proposed coiled coil domain is absent in *A. thaliana* and *Z. mays* (Figure 1A). Taken together these studies suggest that unlike the Rpt1 and Rpt2 motifs of INI1/hSNF5, the region corresponding to amino acids 105–183 of INI1 is absent in yeast and plant SNF5 and has developed into a highly conserved region in vertebrates.

**Figure 2:**
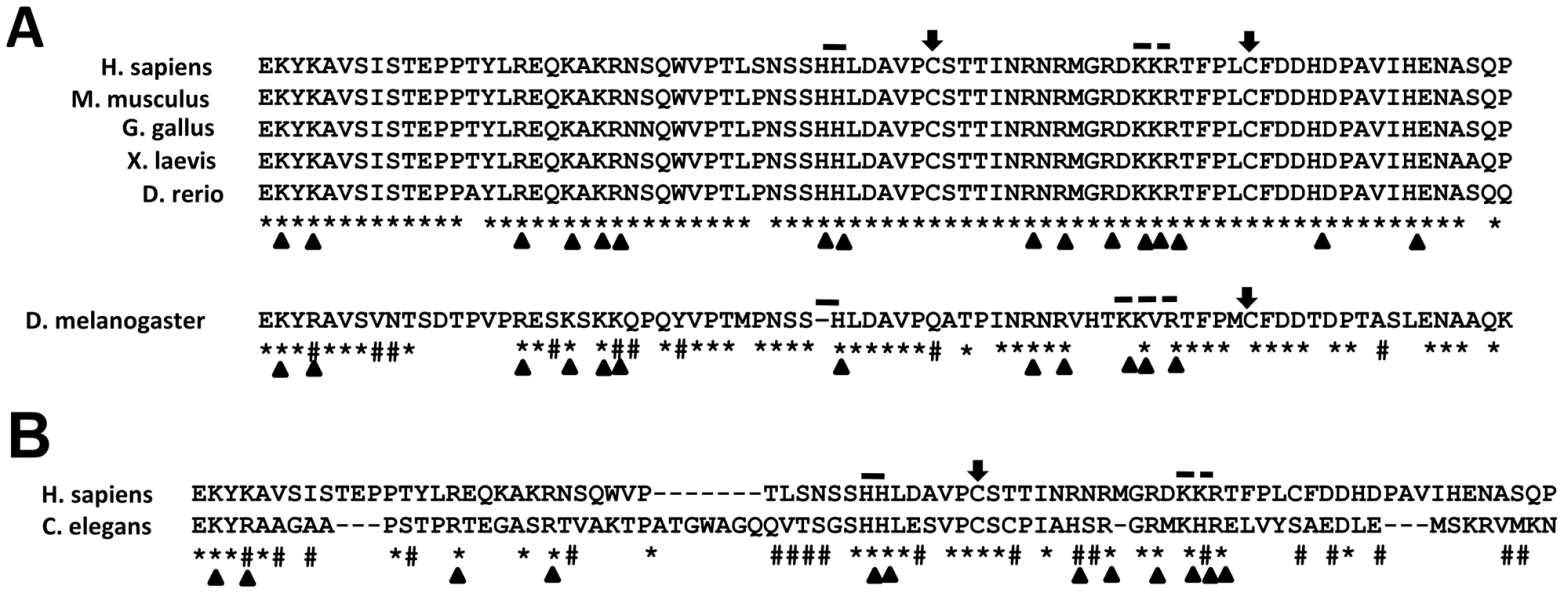
Sequence conservation of the putative DNA binding domain (DBD) of vertebrate INI1/hSNF5, Drosophila SNR1 and *C.* *elegans* SNF5. (A) Sequence comparison of the putative DNA binding domain of Drosophila SNR1 with the domains in vertebrates. Conserved residues are shown by (*) and partially conserved residues by (#). The HH and KKR motifs are shown with dash and broken dash, respectively. The conserved cysteine residues are shown by an arrow. The conserved lysine, arginine and histidine residues are shown with an arrowhead. (B) Sequence comparison of the putative DNA binding domain of *C. elegans* SNF5 with the human domain.

A closer examination of the domain (Figure 2A) in vertebrates shows that the amino acid residues in this region are highly conserved from zebrafish to humans (conserved residues are marked with * and semi-conserved residues are marked with #)). Only four amino acid residues in hINI1 show differences with INI1 from other vertebrates. Drosophila SNR1, which is intermediate between *C. elegans* SNF5 and hINI1, shows about 70% overall homology (54/78 residues of the domain conserved) to hINI1. Positively charged amino acid residues like lysine, arginine and histidine in DNA binding proteins, especially in short motifs of consecutive positively charged residues, contribute to local DNA shape readout e.g minor or major groove binding. When we looked at the conservation of these positively charged residues in vertebrates we found that there are 16 such residues that are highly conserved between the human, mouse, chicken, frog and fish INI1 domains (Figure 2A, arrowhead). Thus, this domain is rich in conserved basic amino acids. However, in Drosophila SNR1 only 12 of these residues are conserved (Figure 2A, arrowhead). Furthermore, SNR1 contains only one histidine residue in place of the conserved di-histidine (HH) motif at positions 141–142 of hINI1 (marked by solid line, Figure 2A) and other vertebrate INI1/SNF5 whereas it contains a valine insertion (KKVR) in place of the conserved KKR motif at position 161–163 in hINI1 (marked by broken line, Figure 2A) and other vertebrate INI1/SNF5. In addition, SNR1 contains only one conserved cysteine residue corresponding to C168 of hINI1 instead of two conserved cysteine residues at position 148 and 168 of hINI1 and other vertebrates (marked by arrow, Figure 2A). Interestingly, *C. elegans* SNF5 which shows 54% overall homology (43/79 amino acids residues conserved) to hINI1 contains 12 conserved positively charged residues, the HH motif is conserved and the KKR motif is replaced by a KHR motif but it has only one conserved cysteine residue corresponding to the cysteine at position 148 of hINI1 (Figure 2B). Furthermore, the *C. elegans* SNF5 domain has gaps in sequence as compared to hINI1 (Figure 2B) and a 7 amino acid insertion in the region corresponding to amino acids 131–132 of hINI1 (Figure 2B).

### The Region 105–183 of INI1 and 90–167 of SNR1 Binds DNA

**Figure 3:**
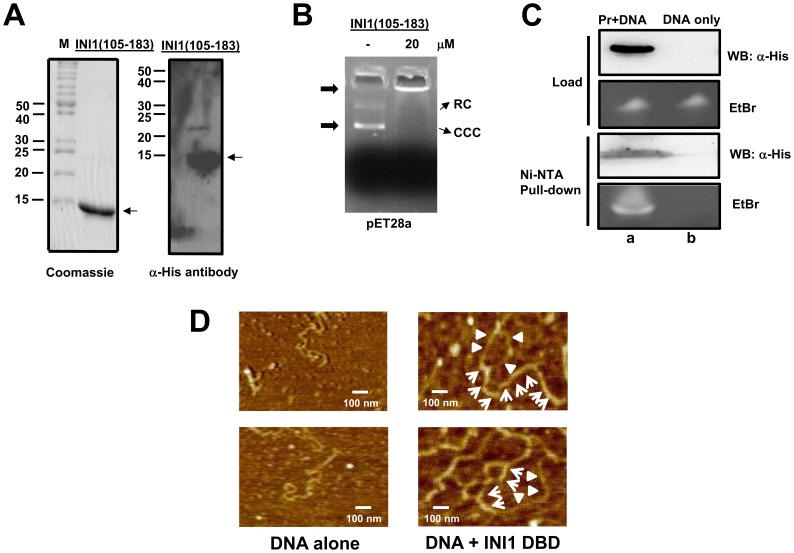
DNA binding studies of INI1 DBD. (A) Coomassie blue stained SDS-PAGE (left panel) and Western blot analysis (right panel) of recombinant, purified INI1 DBD using α-His antibodies as probes. (B) Agarose gel retardation assay (AGRA) of INI1 DBD using 100 ng of pET28a as substrate and indicated amount of polypeptide; RC: relaxed circular DNA, CCC: covalently closed circular DNA. (C) Ni-NTA pull-down assay of INI1 DBD:U5 HIV-1 LTR complex (lane a) and of DNA alone (lane b). The precipitated complex was analyzed by western blot using α-His antibodies as probes to detect INI1 DBD and the co-precipitated DNA was analyzed by ethidium bromide (EtBr) staining following 10% urea-PAGE. The loading controls are shown. (D) Atomic force microscopy (AFM) images of pNEB206A DNA alone (left panel) and in complex with INI1 DBD (right panel). Regions of DNA coated with protein (arrows) and free DNA (arrowhead) is shown.

In order to determine whether INI1(105–183) has DNA binding property, we first cloned the fragment 105–183 from human INI1 and expressed and purified the fragment through a one-step purification process (see Materials and Methods). Due to the nature of cloning, the N-terminus had a 24 amino acid extension in the INI1 fragment. The fragment was purified to ∼95% homogeneity (Figure 3A, left panel) and verified by Western blot analysis (Figure 3A, right panel) using α-His antibodies as probes. The purified INI1(105–183) fragment was tested for its DNA binding property using agarose gel retardation assay (AGRA). At 20 µM protein concentration, the INI1 fragment bound pET28a plasmid efficiently (Figure 3B) in AGRA assay resulting in slower migration of DNA which accumulates near the well. We confirmed that INI1(105–183) contains the minimal DNA binding domain of INI1 by Ni-NTA pull-down assay (Figure 3C) wherein the purified fragment was incubated with the U5 HIV-1 LTR DNA and the complex was precipitated by using Ni-NTA beads. Following washing, the beads containing bound complex were subjected to western blot analysis using α-His antibodies as probes to detect INI1 DBD and 10% urea-PAGE followed by ethidium bromide staining to detect bound DNA (Figure 3C). The loading controls are shown. Ni-NTA-bound His-tagged INI1 DBD was able to efficiently co-precipitate the U5 HIV-1 LTR DNA (Figure 3C, lane a) but DNA alone was not precipitated by the Ni-NTA beads (Figure 3C, lane b) demonstrating that the region amino acids 105–183 comprises the minimal DNA binding domain of INI1. Finally, we confirmed DNA binding by INI1 (105–183) using atomic force microscopy and a linearized plasmid DNA (Figure 3D). The INI1 DBD partially coats the DNA (Figure 3D, right panels, arrows) as compared to free DNA (Figure 3D, left panels) and regions of DNA that are not bound (Figure 3D, right panels, arrowheads) suggesting that the region amino acids 105–183 of INI1 has DNA binding property.

**Figure 4:**
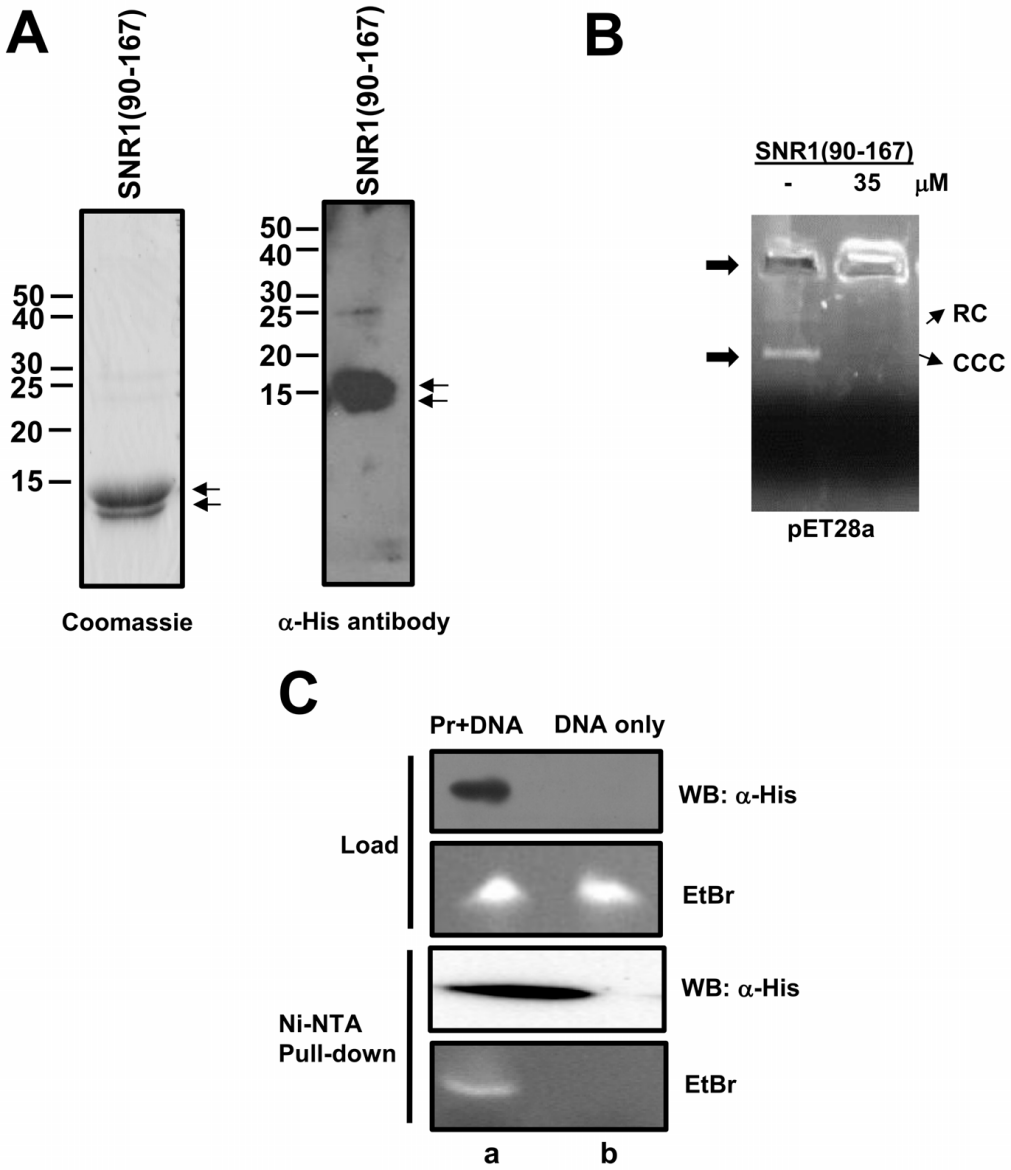
The proposed SNR1 DBD binds DNA. (A) Coomassie blue stained SDS-PAGE (left panel) and Western blot analysis (right panel), with α-His antibodies as probe, of recombinant, purified SNR1 DBD. (B) Agarose Gel Retardation Assay (AGRA) of SNR1 DBD. AGRA assay was carried out using pET28a (undigested, left panel) as substrate and indicated amount of polypeptide; RC: relaxed circular DNA, CCC: covalently closed circular DNA,. (C) Ni-NTA pull-down assays of SNR1 DBD incubated with U5 HIV-1 LTR DNA (lane a). Following washing, the beads containing bound complex were analyzed by western blot using α-His antibodies as probes to detect SNR1 DBD and 10% urea-PAGE followed by ethidium bromide staining to detect bound DNA. DNA only control (lane b) was processed and analyzed in the same manner. The loading controls are shown.

In order to characterize the corresponding region of Drosophila SNR1, we cloned, expressed and purified SNR1(90–167) through a one-step purification process (see Materials and Methods). Due to the nature of cloning, the N-terminus had a 37 amino acid extension in SNR1. The fragment was purified to ∼95% homogeneity (Figure 4A, left panel) and verified by Western blot (Figure 4A, right panel) using α-His antibodies as probes. The SNR1 fragment migrates as a doublet perhaps as a result of proteolytic cleavage at the C-terminal end or due to the effect of detergent solubilization as has been previously observed with the full-length INI1 protein [10]. The DNA binding property of SNR1(90–167) was tested by agarose gel retardation assay (AGRA). The SNR1 fragment binds pET28a plasmid (Figure 4B) efficiently at 35 µM protein concentration. The bound DNA shows slower mobility and accumulates near the well. The SNR1(90–167) fragment was then tested for its ability to bind a short double stranded DNA in a Ni-NTA pull-down experiment (Figure 4C). As INI1 binds to DNA non-specifically [10], we used the LTR DNA itself to test DNA binding by SNR1(90–167). DNA was incubated alone or with purified, recombinant SNR1(90–167) for 30 min and then precipitated with Ni-NTA beads. The washed beads containing bound complex were analyzed by western blot analysis using α-His antibodies as probes to detect SNR1 DBD and 10% urea-PAGE followed by ethidium bromide staining to detect bound DNA (Figure 4C). While SNR1(90–167) was able to efficiently co-precipitate DNA, DNA alone was not precipitated under these conditions (Figure 4C). These studies show that the region amino acids 90–167 in SNR1, indeed has DNA binding activity similar to INI1 DBD. Taken together, these studies demonstrate that the region in INI1/SNF5 family of proteins corresponding to amino acids 105–183 of human INI1 comprises the minimal DNA binding domain of this family of proteins.

The full-length recombinant INI1 protein is only moderately soluble and the purified protein is obtained in low amount (nM range) [10] making it hard to study its properties in biochemical and biophysical assays requiring significant amounts of protein. On the other hand, the fragment INI1(105–183) is obtained in micromolar amount after purification. Therefore, in order to further investigate the DNA binding properties of INI1, we used the purified DBD of INI1 in our studies.

### Stoichiometry of INI1 DBD:DNA Complex

**Figure 5:**
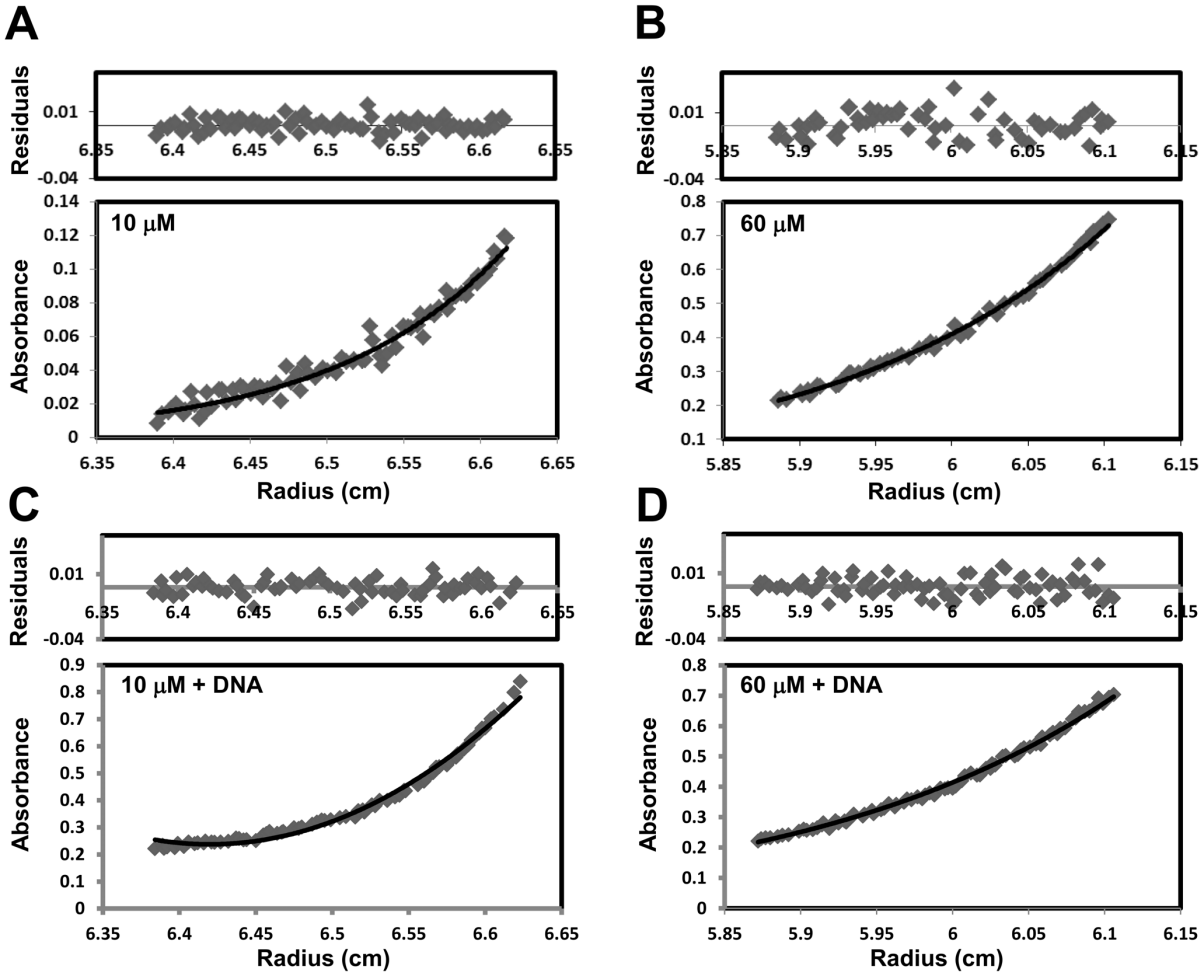
Sedimentation equilibrium analysis of INI1 DBD and INI1:DNA complex. (A) Representative sedimentation equilibrium (SE) profiles of 10 µM (A) and 60 µM (B) of INI1 DBD generated from data collected at 280 nm. Representative sedimentation equilibrium (SE) profiles of a reaction mixture of 10 µM INI1 DBD and 10 µM U5 HIV-1 LTR DNA diluted 1∶7.5 x with buffer (C) and a reaction mixture of 60 µM INI1 DBD and 60 µM U5 HIV-1 LTR DNA diluted 1∶40 x with buffer (D) generated from data collected at 260 nm. Lower panels: Radial distribution of the concentration of INI1 DBD (A and B) and DNA (C and D) both free and in complex with INI1 DBD at sedimentation equilibrium. The solid line represents best fit. Upper panels: Distributions of the residuals around a zero mean.

Since previous studies have shown that full-length wild-type INI1 is a multimer [10] and that the region 1–141 may enhance multimerization [10], the fragment INI1 (105–183) was next subjected to sedimentation equilibrium analysis at different protein concentrations to determine its multimerization status. This technique allows direct determination of absolute molecular mass independent of protein shape. Furthermore, compared to analytical gel filtration chromatography, sedimentation equilibrium analysis is a preferred technique to study protein-DNA complexes and determine stoichiometry of binding from molecular mass as it is not affected by the non-globular nature of DNA. Sedimentation equilibrium runs were performed at two different protein concentrations (10 µM and 60 µM) of the INI1 DBD and the absorbance was monitored at 280 nm. Each data set was analyzed separately (see Materials and Methods). Global fitting of the data sets (using SEDPHAT and the species analysis parameter) yielded an estimated molecular mass of 13372±37 Da at 10 µM protein concentration and 21862±206 Da at 60 µM protein concentration (Table 1). Figures 5A and 5B show the non-linear least squares fits of the data for 10 µM and 60 µM INI1 DBD, respectively. The calculated molecular weight of the purified INI1 DBD fragment is 11631.9 Da (Table 1). Thus, the experimental molecular mass of INI1 DBD at 10 µM and 60 µM protein concentrations is about 1.15 and 1.88 times the molecular weight of INI1 DBD, respectively, suggesting that at low concentration the DBD is a monomer and at high concentration it is a dimer. The slightly higher molecular mass of the monomer and slightly lower molecular mass of the dimer may be due to dynamic association-dissociation of a fraction of the monomer-dimer protein during centrifugation.

**Table 1:** Determination of stoichiometries of INI1 DBD binding to U5 HIV-1 LTR DNA from analytical ultracentrifugation studies.

Macromolecular components	Calculated MW^a^ (daltons)	Estimated average mass*^b^* (daltons)	Proposed composition of complex
INI1 DBD	11631.9	_	_
U5 HIV-1 LTR DNA	13613	_	_
INI1 DBD (10 µM)	_	13372±37	Monomer (1.15 x MW)
INI1 DBD (60 µM)	_	21862±206	Dimer (1.88 x MW)
INI1 DBD (10 µM) +10 µM DNA	_	15422±1438; 25415±510; 36287±129	(Free DNA)+(Monomer +1 DNA)+(2 Monomer +1 DNA)
INI1 DBD (60 µM) +60 µM DNA	_	16628±1193; 36992±504; 50281±769	(Free DNA)+(Dimer +1 DNA)+(Dimer +2 DNA)

a Calculated from amino acid or nucleotide composition, *^b^*Based upon sedimentation equilibrium data, ± = SD (standard deviation).

Next, we carried out sedimentation equilibrium runs (centrifuged following dilution) of protein-DNA complexes formed by incubating (i) 10 µM INI1 DBD with 10 µM U5 HIV-1 LTR DNA, and (ii) 60 µM INI1 DBD with 60 µM U5 HIV-1 LTR DNA, followed by monitoring the absorbance at 260 nm (at the dilution used there is very little interference at 260 nm from the protein). A global fit of the data (as described above) yielded molecular masses of 15422±1438 Da, 25415±510 Da and 36287±129 Da for the protein-DNA complexes formed in (i), and 16628±1193 Da, 36992±504 Da and 50281±769 Da in (ii) (Table 1). Figures 5C and 5D show the non-linear least squares fits of the data for reaction mixtures (i) and (ii), respectively. The calculated molecular weight of the U5 HIV-1 LTR DNA is 13613 Da. At low protein concentration (i), the molecular mass 25415±510 Da corresponds to one molecule of the DBD monomer bound to one molecule of the DNA whereas the molecular mass 36287±129 Da corresponds to two molecules of the monomer bound to one molecule of DNA (Table 1). At high protein concentration (ii), the molecular mass 36992±504 Da corresponds to one molecule of the dimer bound to one molecule of DNA and the molecular mass 50281±769 Da corresponds to one molecule of dimer bound to two molecules of DNA (Table 1). The molecular masses 15422±1438 Da (i) and 16628±1193 Da (ii) correspond to the free DNA. The higher molecular mass of free DNA is due to the fact that the partial specific volume of the protein-DNA complex (0.6478 ml/gm and 0.6691 ml/gm), rather than DNA alone (0.59 ml/gm), was used to fit the data. In summary, our data suggests that at low protein concentration the INI1 DBD monomer binds to DNA with a stoichiometry of 2∶1 (protein:DNA) and at high protein concentration the INI1 DBD dimer binds to DNA with a stoichiometry of 1∶2 (protein:DNA).

### Isothermal Calorimetry of INI1 DBD Binding to DNA

**Figure 6:**
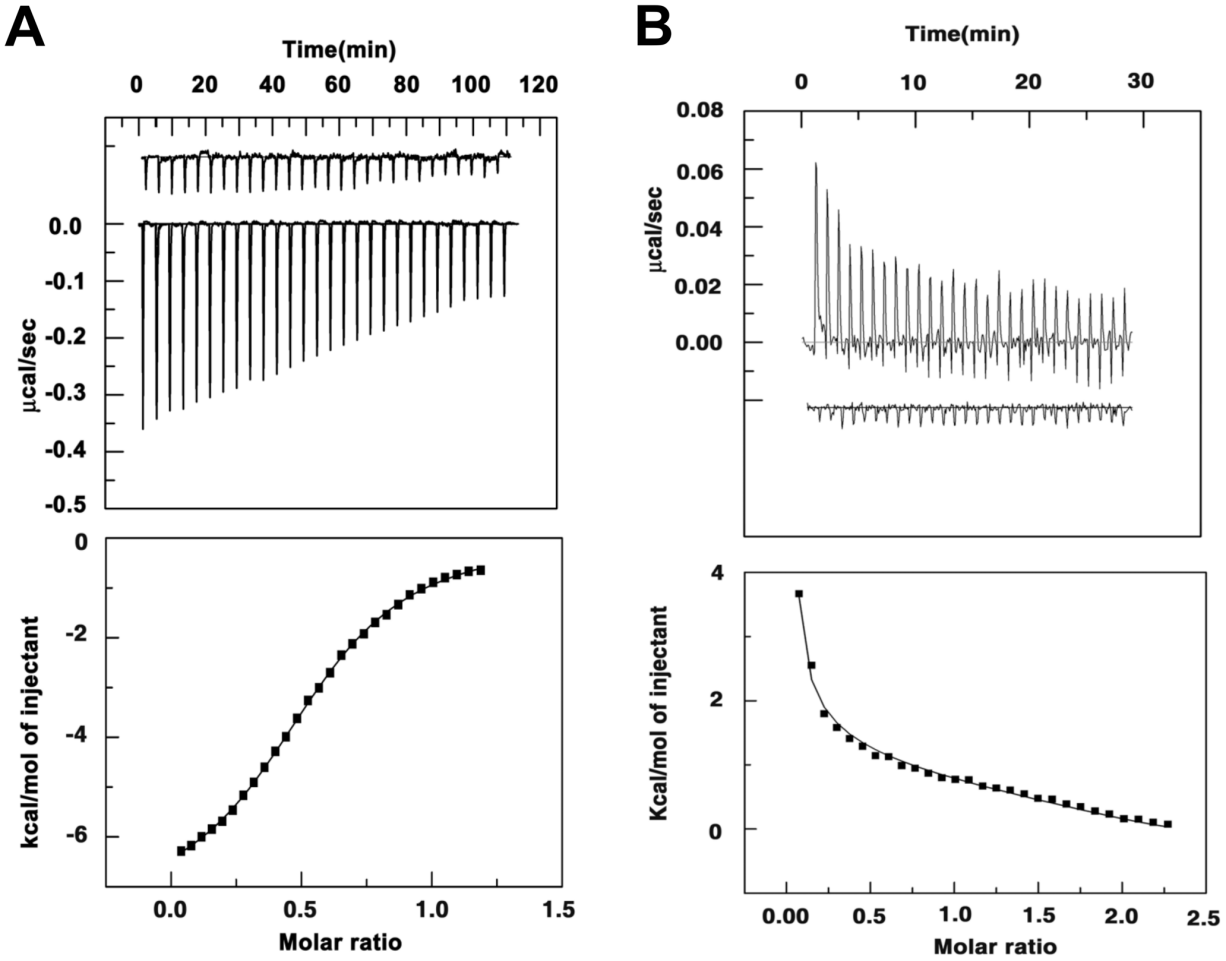
Isothermal calorimetry. ITC of INI1 DBD binding to U5 HIV-1 LTR DNA using 10 µM INI1 DBD (monomer) and 55 µM U5 HIV-1 LTR DNA (A) and 30 µM INI1 DBD (dimer) and 325 µM U5 HIV-1 LTR DNA (B). Top panel: Raw data of heat associated with mixing of DNA and protein. Heat of dilution is shown in the inset (not to scale). Bottom panel: Heat associated with each injection is obtained by integration of the area under the peak as a function of time. Binding curve (fitted) obtained by subtracting heat of dilution from heat associated with mixing of protein and DNA. Data is representative of multiple experiments.

Isothermal calorimetry (ITC) is a powerful technique for monitoring protein-DNA binding. Not only does it allow precise measurement of dissociations constants, but also it allows simultaneous measurement of the entropy and enthalpy changes associated with the binding process. ITC experiments were carried out using purified recombinant INI1 DBD and the U5 HIV-1 LTR DNA (Figures 6A and 6B) at two different protein concentrations (10 µM of monomer and 30 µM of dimer). The DNA concentration in the syringe was kept at 5.5 x and 11 x the concentration of 10 µM of monomer and 30 µM of dimer, respectively. The raw and fitted data are shown in Figures 6A and 6B, respectively. Data was fitted with different binding models *viz* one-site, two-site and sequential binding sites. At a protein concentration of 10 µM of monomer, the DNA binding reaction was exothermic and could be fitted with the one-site binding model yielding K_d_  = 0.94±0.02 µM, with a large enthalpy change (ΔH = –29.95±0.57 KJ/mole) and only nominal entropy change (TΔS  = 4.43±0.78 KJ/mole) (Table 2). The Gibb’s free energy change ΔG is –34.38±0.16 KJ/mole and the stoichiometry (n) is 0.49±0.04 (Table 2). It should be noted here that the stoichiometry of binding obtained from ITC data is in agreement with our observation from analytical ultracentrifugation experiments that two molecules of the INI1 DBD monomer binds to one molecule of the U5 HIV-1 LTR DNA. However, at a protein concentration of 30 µM of dimer, the DNA binding reaction could be best fitted with a sequential binding model where the number of binding sites was two with the first binding event being endothermic and the second binding event being exothermic. The data yielded poor fits with other binding models. The binding constants obtained for the sequential model were K’_d1_ = 222±51.8 µM and K’_d2_ = 1.16±0.31 µM (Table 2). Although the Gibb’s free energy change for the first (ΔG’_1_ = –21.13±0.64 KJ/mole) and the second (ΔG’_2_ = –30.65±1.9 KJ/mole) binding reactions differ nominally (∼9 KJ/mole), the enthalpy and entropy contributions to the two Gibb’s free energy terms are different. The first binding event is associated with a positive enthalpy change (ΔH’_1_ = 115.67±29.7 KJ/mole) that is compensated by a large positive entropy change (TΔS’_1_ = 136.8±33.7 KJ/mole) leading to a negative Gibb’s free energy change (ΔG’_1_ = –21.13±0.64 KJ/mole) (Table 2). On the other hand, the second binding event is associated with a negative entropy change (TΔS’_2_ = –75.7±32.2 KJ/mole) that is compensated by a negative enthalpy change (ΔH’_2_ = –106.3±30.2 KJ/mole) leading to a negative Gibb’s free energy change (ΔG’_2_ =  −30.65±1.9 KJ/mole) (Table 2). Thus, INI1 DBD monomer and dimer binding to DNA show different stoichiometries of binding, binding affinities and thermodynamic parameters. The significance of these findings is discussed.

**Table 2:** Determination of stoichiometries, binding constants and thermodynamic parameters of INI1 DBD-DNA binding from isothermal calorimetry studies.

Reaction components	N	K_d_ (µM)	ΔH (KJ/mole)	TΔS (KJ/mole)	ΔG (KJ/mole)
INI1 DBD monomer (10 µM) +55 µM DNA	0.49±0.04	0.94±0.02	−29.95±0.57	4.43±0.78	−34.38±0.16
INI1 DBD dimer (30 µM) +325 µM DNA	_	(i) 222±51.8 (ii) 1.16±0.31	(i) 115.67±29.7 (ii) −106.3±30.2	(i) 136.8±33.7 (ii) −75.7±32.2	(i) −21.13±0.64 (ii) –30.65±1.9

N = stoichiometry, ± = SE (standard error).

## Discussion

INI1/hSNF5 is a component of transcription complexes like the chromatin remodeling hSWI/SNF transcription activator complex and the Sin3A-HDAC1 transcription repressor complex. Apart from transcription regulation, INI1 has been implicated in cell proliferation and survival, mitosis, DNA repair and cell migration [7], [18], [19], [20], [21]. INI1 represses basal transcription of the HIV-1 promoter [22] and also modulates the expression of different cellular genes [19]. However, it is not known whether INI1 mediates its effect on transcription and other DNA-dependent processes by virtue of its ability to bind DNA. Furthermore, mutation or lack of INI1 causes the pediatric cancer ATRT (Atypical terratoid and rhabdoid tumor) and INI1 is also one of the host factors that play an important role in multiple steps of the HIV-1 life cycle. Repression of cyclin D1 expression, by INI1, is a key pathway in the genesis of rhabdoid cancers [7], [8]. However, the possibility that other functions of INI1 may play a role in the genesis of rhabdoid cancers cannot be ruled out. The INI1 polypeptide contains two motifs, Rpt1 and Rpt2, which are involved in protein-protein interaction and also contains a masked NES (nuclear export sequence). INI1 binds to DNA non-specifically, at the minor groove [10]. It also appears that DNA binding by INI1 may be important for its ability to stimulate the activity of HIV-1 integrase [10]. Thus, it is important to investigate the DNA binding property of INI1 in order to determine its biological role in INI1 function.

We have found that the proposed DNA binding region of INI1 (amino acids 105–183) is not present in lower eukaryotes like the yeast *S. cerevisiae*, and plants like *A. thaliana*. It is present in *C. elegans* and has evolved into a highly conserved region in vertebrates. This region of INI1 is sufficient to bind DNA, clearly showing that amino acid 105–183 of INI1 comprises the DNA binding domain of INI1. We further show that the corresponding region in the Drosophila homologue SNR1 (amino acids 90–167) is sufficient to bind DNA demonstrating that the DNA binding property of this region in the INI1/SNF5 family is conserved. It will be important to study how the DBD of Drosophila SNR1 and putative DBD of *C. elegans* SNF5, which show 58% and 30% identity to human INI1 DBD, respectively, behave with respect to their DNA binding property as compared to the human domain. This will provide us with insights into how the function of this domain has evolved. At this point we do not rule out the possibility that, in lower eukaryotes and plants, SNF5 uses other regions of the polypeptide to bind DNA.

**Figure 7:**
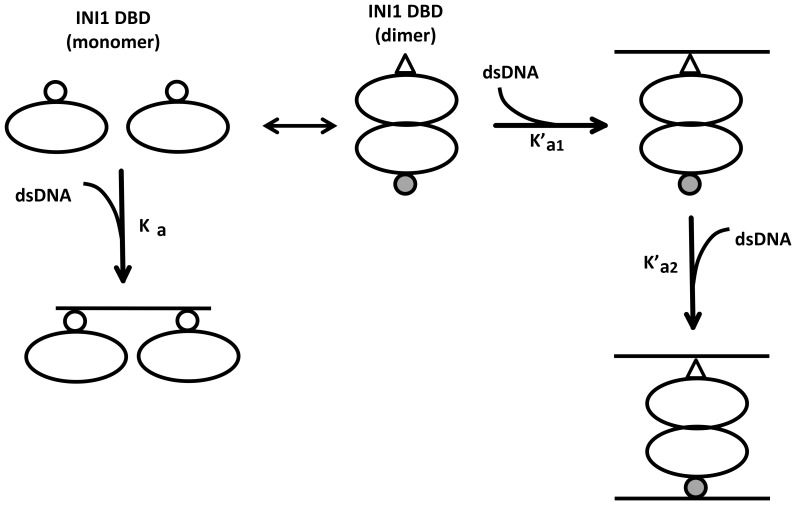
Model showing different modes of INI1 DNA binding. The INI1 DBD undergoes concentration dependent multimerization. Two molecules of monomeric DBD binds to one molecule of U5 HIV-1 LTR DNA whereas one molecule of dimeric DBD binds to two molecules of U5 HIV-1 LTR DNA.

The purified, recombinant, full-length INI1 protein is sparingly soluble and is obtained only in nanomolar amounts. On the other hand, the purified, recombinant INI1 DBD is obtained in micromolar amounts. This prompted us to use this fragment to study INI1 DNA binding properties. We first tested the fragment for its multimerization property as the full-length protein is a multimer [10]. Multimerization of INI1 is concentration-dependent [10]. Furthermore, deletion of the region, amino acids 1–141, of INI1, overlapping the DBD, reduces the affinity of N-terminal fragments of INI1 fused to GAL4AD for GAL4DBD-INI1 (WT) in yeast two-hybrid assays [10]. This suggests that the N-terminal region of INI1 may contribute to its multimerization in addition to the previous findings that a region containing the Rpt1 and Rpt2 motifs comprises the minimal multimerization domain [10]. Analytical ultracentrifugation of INI1 DBD showed that at low protein concentration the INI1 DBD behaves as a monomer but at high protein concentration it is a dimer (Figure 7). In addition, we determined the stoichiometries at which INI1-DNA complexes are formed at different protein concentrations. We found that the monomer binds DNA at a protein:DNA ratio of 2∶1 i.e. two monomers are bound to each molecule of the 22/23 nt long ds U5 HIV-1 LTR DNA (Figure 7). On the other hand, the dimer binds the same DNA at a protein:DNA ratio of 1∶2 i.e. each dimer binds to two molecules of DNA (Figure 7). Isothermal calorimetry experiments confirmed that the monomer binds to the LTR DNA at one site with a stoichiometry (N) of ∼0.5. The binding reaction is exothermic and enthalpy driven with a dissociation constant of ∼1 µM. However, the dimer binds sequentially to two DNA molecules with the first binding constant (K_d2_ = 222 µM) two orders of magnitude higher than the second binding constant (K_d3_ = 1.16 µM). The first binding event is endothermic and entropy driven whereas the second binding event is exothermic and enthalpy driven. Thus, the binding of DNA by the monomeric form is different from that of the dimeric form. Based on these findings we hypothesize that upon dimerization the DBD undergoes a conformational change in the DNA binding pocket. Given the two orders of magnitude increase in the dissociation constant of the first binding event by the dimer, as compared to the monomer, it is likely that the binding to the first DNA molecule by the dimer is less preferred than DNA binding by the monomer. However, once the dimeric DBD is bound to the first DNA molecule, the second binding event becomes easier leading to a two order of magnitude decrease in the dissociation constant.

Protein-DNA interactions are vital to the progress of biological processes such as transcription, replication, recombination and repair. The results presented in this paper provide insights into the possible mechanism by which INI1 may stimulate HIV-1 integrase (IN) activity. Previously, it has been demonstrated that INI1 stimulates IN activity at low IN concentration and low INI1:IN ratio and inhibits IN activity at high IN concentration and high INI1:IN ratio [10]. Multimerization of INI1 is concentration-dependent and essential for its ability to bind HIV-1 IN efficiently and inhibit IN activity [10]. On the other hand, DNA binding by INI1 may be important for its ability to stimulate IN activity [10]. Our finding, that the INI1 DBD undergoes concentration-dependent multimerization and that the DBD monomer and dimer show different DNA binding properties, provides important clues as to how INI1 may switch from stimulating INI1 activity to inhibiting it. We hypothesize that there are two competing multimerization-dependent functions of INI1 *viz* efficient binding to IN and alternate DNA binding properties. The multimerization of the DBD reduces the binding affinity of the first binding event with DNA by two orders of magnitude as compared to the monomer. On the other hand, multimerization of INI1 promotes IN-binding. This raises the possibility that multimerization of INI1 disfavors its DNA-dependent function e.g. stimulation while favoring INI1-IN interaction which leads to inhibition. Our future work will focus on addressing this hypothesis.
